# The significance of trained immunity in cancer

**DOI:** 10.3389/fimmu.2025.1665099

**Published:** 2025-12-01

**Authors:** Junxing Qu, Xinya Guo, Xinru Wang, Huiwen Meng, Peizhi Li, Zhiheng Sun

**Affiliations:** 1 Institutes of Health Central Plains, Henan Medical University, Xinxiang, Henan, China; 2 Xinxiang Key Laboratory for Tumor Drug Screening and Targeted Therapy, Xinxiang, Henan, China; 3 College of Life Science, Institute of Biomedical Science, Henan Normal University, Xinxiang, Henan, China; 4 State Key Laboratory of Cell Differentiation and Regulation, Xinxiang, Henan,, China; 5 Department of Anesthesiology, Xinxiang First People’s Hospital, The Affiliated People’s Hospital of Xinxiang Medical University, Xinxiang, Henan, China

**Keywords:** trained immunity, lung cancer, gastric cancer, liver cancer, colorectal cancer

## Abstract

Trained immunity (TI) represented a unique state of innate immune activation, characterized primarily by persistent epigenetic modifications in immune cells. This phenomenon was first observed during pathogen infections and vaccinations, where it manifested as enhanced defensive responses in innate immune effector cells—such as those of the mononuclear phagocyte system and natural killer cells—upon re-stimulation. Cancer was a disease with complex mechanisms, marked by the loss of normal growth regulation in cells due to genetic mutations or epigenetic dysregulation, leading to abnormal proliferation and dissemination. With hundreds of subtypes, cancer could arise in virtually any human tissue or organ. The primary cause of cancer-related mortality was metastasis, which referred to the spread of cancer cells from their original site to distant organs and accounted for approximately 90% of cancer deaths worldwide. The induction of TI involved multiple immune components including myeloid cells, natural killer cells, pattern recognition receptors, and various cytokines. Notably, the enhanced response observed during secondary stimulation remained non-specific to particular pathogens. Compared to conventional therapeutic approaches, TI demonstrated superior systemic immune activation. Simple pharmacological stimuli such as β-glucan or Bacillus Calmette-Guérin (BCG) not only triggered innate immune responses but also conferred benefits to adaptive immunity, resulting in more rapid immune activation and enhanced efficacy. TI enhanced the capacity of immune cells to recognize and eliminate cancer cells, playing a critical role in countering metastasis. In this review, we summarized existing knowledge in the field, focusing on the mechanisms underlying TI induction and its significance in combating cancer.

## 1 Introduction

The immune system was classically divided into two categories (1): innate immunity and acquired immunity. Innate immunity, which developed through long-term evolutionary processes, was present from birth and provided broad-spectrum, non-specific protection against pathogens. In contrast, acquired immunity represented an antigen-specific response that was individualized, non-heritable, and characterized by immune memory. TI described the epigenetic reprogramming of bone marrow progenitor cells induced by infection or vaccination, which conferred enhanced non-specific protection against secondary infections (2).This phenomenon differed fundamentally from classical immune memory in several aspects. First, TI primarily involved innate immune components including bone marrow progenitor cells, natural killer cells (NK cells), innate lymphoid cells (ILCs), and pattern recognition receptors, rather than the adaptive immune cells characteristic of classical memory responses. Second, TI-mediated responses to secondary challenges were not antigen-specific but rather driven by transcriptional regulation and epigenetic modifications. Furthermore, the functional enhancements in innate immune cells following TI typically persisted for weeks to months after clearance of the initial stimulus, in contrast to the years-long persistence of classical immune memory (3).The mechanistic foundation of TI was closely associated with metabolic reprogramming in immune cells (4). This process could occur either in bone marrow progenitor cells (central TI) or in terminally differentiated innate immune cells and peripheral tissues (peripheral TI) (5).TI played significant roles in both disease prevention and treatment strategies. For example, it enhanced vaccine efficacy and offered novel therapeutic opportunities for cancer and inflammatory disorders. Among innate immune cells, neutrophils emerged as crucial mediators of TI. As the most abundant innate immune population derived from bone marrow, neutrophils served as primary responders to infectious or inflammatory insults (6).Induction of TI in lung-resident alveolar macrophages (AMs) enhanced their capacity for cellular debris clearance and tissue repair (7). However, TI could also contribute to disease pathogenesis in certain contexts. In chronic metabolic disorders, autoimmune conditions, and neurological diseases, TI might promote persistent or maladaptive immune activation. Under these circumstances, TI represented a potential driver of disease progression through dysregulation of innate immune responses (8).

Cancer represents a class of malignant tumors whose incidence has been rising steadily in the 21st century. Current statistics indicate approximately one in four individuals faces cancer risk (9). In 2020, cancer claimed nearly 600,000 American lives. While children and young adults demonstrate relatively low cancer incidence and mortality rates, these figures increase exponentially with age after adulthood. Among adults, lung, colorectal, and breast cancers account for the highest diagnosis rates (10). Emerging evidence suggests a strong correlation between cancer incidence and obesity, with data indicating overweight conditions elevate risk for at least 13 cancer types, including esophageal, pancreatic, colorectal, and thyroid cancers (11). Cancer research faces significant challenges due to intertumoral heterogeneity and dynamic cellular characteristics, complicating the development of effective treatments (12). Notably, cancer cells exhibit substantial signaling pathway alterations that profoundly impact cellular growth, proliferation, metastasis, and apoptosis. These pathway changes coincide with metabolic reprogramming, where both proto-oncogenes and tumor suppressor genes exert substantial metabolic influence (13).Current therapeutic research explores multiple avenues. Epigenetic modulation has emerged as a potential intervention strategy by targeting epigenetic genes involved in carcinogenesis (14). Existing medications like metformin have demonstrated cancer risk reduction (15). Certain vaccines, including recombinant BCG, have shown capacity to enhance immunity by upregulating pro-inflammatory factors, inducing epigenetic and metabolomic changes, and improving both immune function and anti-tumor efficacy (16). Growing evidence indicates that TI system generated anti-tumor responses (17).

TI enhanced pathogen resistance and strengthened immune surveillance. Research showed that induced TI activated immune cells effectively. The induction of TI in cancer contexts may represent a novel therapeutic approach (5). This review summarizes various cancer types and mechanisms while analyzing the role of TI in oncogenesis, potentially offering new perspectives for cancer treatment strategies.

## 2 Trained immunity

The body’s immune system consists of two main types: innate immunity and acquired immunity (18). Phylogenetically, lower animals possessed only innate immunity while vertebrates developed specific immunity. The innate immune system comprised barrier structures, innate immune cells and innate immune molecules. Barrier structures included three types: interspecific barriers, skin and mucous membrane barriers, and internal barriers. The interspecific barrier, also known as species immunity, represented genetic resistance of certain species to specific pathogens (19). The skin and mucous membrane barrier formed from external body coverings and cavity linings (20), while internal barriers included the blood-brain barrier (21), blood-fetal barrier and blood-thymus barrier. Key innate immune molecules involved defensins, complement system, cytokines, lysozyme and other effector factors. Innate immune cells consisted of phagocytes, innate lymphoid cells, natural killer cells, and dendritic cells. The recognition mechanism of innate immunity differed from adaptive immunity by targeting conserved microbial patterns rather than specific antigens, exhibiting relatively limited specificity (22). Adaptive immune responses were MHC-restricted and involved coordinated actions of various immune cells and molecules, with antigen specificity being their most distinctive feature (23).

The concept of TI emerged when researchers discovered that innate immunity could enhance protection against reinfection in organisms lacking adaptive immunity, challenging the conventional view that only adaptive immunity could establish immune memory (24). This innate immune memory phenomenon was termed TI, which was mediated through epigenetic reprogramming. Unlike mutations or genetic recombination in adaptive immunity, TI induced transient changes in gene expression and cellular physiology without permanent genetic alterations (3).

The molecular basis of TI involved metabolic rewiring, where epigenetic modifications interacted with cellular metabolism to regulate gene transcription (25). When occurring in bone marrow precursors of innate immune cells, it was called central TI, while peripheral TI referred to changes in tissue-resident immune or non-immune cells (5).

### 2.1 TI and monocytes

In TI, monocytes serve as crucial functional effector cells. Their involvement in TI is primarily manifested through their epigenetic features, functional mechanisms, regulatory pathways, and long-term *in vivo* memory effects. A key characteristic of trained monocytes is the lactylation (H3K18la) of the lysine residue at position 18 on histone H3. This modification predominantly occurs in distal regulatory regions and exhibits a positive correlation with both active chromatin states and gene transcription. Notably, it persists even after the removal of the training stimulus. Moreover, when monocytes encounter a secondary stimulus, H3K18la is closely linked to the transcription of “training-associated” genes—ultimately sustaining the enhanced response traits of the TI system. From a regulatory mechanism perspective, the elevated lactic acid production induced during TI promotes the generation of pro-inflammatory cytokines, a process that depends on histone lactylation. If lactic acid production or histone lactylation is pharmacologically inhibited, the TI response is abrogated. Additionally, genetic polymorphisms in LDHA (a gene involved in lactic acid production) and EP300 (a gene potentially involved in lactic acid regulation) can also modulate the efficacy of TI. At the *in vivo* level, following BCG vaccination, histone lactylation modifications associated with monocyte function persist in the body for 90 days. This finding not only confirms that H3K18la is an epigenetic marker of innate immune memory, but also underscores the role of monocytes in the long-term maintenance of TI (26) (**Table 1**). Studies have shown that following Plasmodium infection, its metabolic product hemozoin (Hz) accumulates continuously in the bone marrow, a process that can induce TI. Specifically, Hz enhances the accessibility of granulocyte-macrophage progenitors (GMPs) via the MyD88-dependent pathway, thereby laying the groundwork for monocyte generation and functional remodeling. In mice that have recovered from Plasmodium infection, the number of Ly6C^+^ monocytes in the peripheral blood is significantly increased—and this elevated count can be sustained for up to 6 months post-infection. Additionally, certain distinct monocyte subtypes emerge during this period. Notably, the recovered mice exhibit enhanced functional capacities of these monocytes, including increased reactive oxygen species (ROS) production, as well as improved bactericidal and phagocytic activities (27) (**Table 1**).When human monocytes are stimulated with β-G, BCG, or Candida albicans, histone modifications—including H3K4me1 and H3K4me3—occur in the promoter regions of inflammatory cytokine genes. These modifications collectively form the epigenetic signatures underlying TI. Following training, human monocytes undergo alterations in cellular metabolic pathways, which are characterized by a marked increase in glycolysis, glutamine catabolism, and cholesterol biosynthesis (28) (**Table 1**). In studies where a TI model was established via BCG inoculation, monocyte-like THP-1 cells were employed as a research subject to mimic the characteristics of monocytes. Transcriptome analysis revealed that the Akt-HIF-mTOR signaling pathway was activated in these cells (29) (**Table 1**). The TI of the human body causes epigenetic reprogramming of monocytes, presenting an enhanced and persistent pro-inflammatory phenotype. This phenotype can provide non-specific protection for the body and help resist reinfection (30).This study identified a combination therapy involving β-G-induced TI and irreversible electroporation (IRE) enhancement, confirming that monocytes serve as the core effector cells of TI. In the mechanism of action: First, β-G acts as the core inducer and is recognized by monocytes via intraperitoneal injection (IP) or oral administration. The Dectin-1 receptor on the monocyte surface is the primary binding target for β-G; upon their binding, downstream signaling cascades are triggered. Subsequently, IRE releases damage-associated molecular patterns (DAMPs), which function as “secondary stimuli.” These DAMPs bind to the surface receptors of monocytes that have already been trained by β-G, significantly boosting the trained response of the monocytes. Notably, the intensity of this response increases in a dose-dependent manner with the elevation of the secondary stimulus concentration (31) (**Table 1**). Monocytes and macrophages are the main cell populations in trained immunity research, and more recent advances are most likely to come from a focus on monocytes.

**Table 1:** The impact of diverse stimuli on trained immunity for tumor combat.

Stimulant	Stimulate cell groups	Influence	Reference
BCG	Monocyte	Histone lactation promotes the production of pro-inflammatory cytokines	(26)
BCG	THP-1 cells	The Akt-HIF-mTOR signaling pathway is activated	(29)
BCG	Alveolar mucosal macrophages	Intravenous injection can enhance the anti-tumor activity of immune checkpoint blockade	(74)
BCG	Macrophage	It can increase the proportion of CTL and inhibit the growth of bladder tumors	(109)
BCG	Dendritic cells	Increased antigen uptake and presentation promote the proliferation of CTL and inhibit the progression of bladder cancer	(109)
β-G	TI	The tumor burden of pancreatic cancer in the mouse model was significantly reduced	(31)
BCG is used in combination with β-G	In situ bladder cancer	Reprogrammed neutrophils exhibited enhanced ROS production, leading to tumor vascularization and reduced growth	(109)
BCG combined with molecular intervention	Macrophage	Polarizing macrophages into the anti-tumor M1 phenotype ultimately led to a significant increase in apoptosis of bladder cancer cells	(110)
GPG	Tregs	Reverse immunosuppressive TME and MDSCs to inhibit the development of breast cancer	(102)
Hz	Monocyte	The MyD88-dependent pathway enhances the accessibility of GMPs and lays the foundation for monocyte generation	(27)
Hz	Monocyte	The ROS in mouse monocytes increased, and the bactericidal and phagocytic activities were elevated	(27)
LNT	Tumor suppressor factor	Activate the tumor suppressor factor p53 to exert an inhibitory effect on cervical cancer	(91)
WGP	Macrophage	Reprogram macrophages to enhance their reactivity to LPS and reduce melanoma metastasis	(68)
β-G	Neutrophils	Inhibit the growth of lung tumors through ROS-dependent mechanisms	(73)
β-G nanoplatform	Tumor cells	It significantly induces oxidative stress, aggravates DNA damage in breast cancer cells, and thereby enhances the anti-cancer effect	(105)
β-G/BCG/ Candida albicans	Human monocytes	Histone modifications in the promoter regions of inflammatory cytokines such as H3K4me1 and H3K4me3	(28)
β-G nanoformulations	Lymph nodes and dendritic cells	It significantly promotes the antigen uptake and maturation of DC, accompanied by an increase in the production of pro-inflammatory cytokines	(77)
β-G can be administered by intraperitoneal injection or orally	Monocyte	The Dectin-1 receptor is the main binding target. After binding, the downstream signal cascade is triggered	(31)
Intravenous injection of BCG	CTL	Promote the proportion of CTL cells and enhance the anti-tumor bladder microenvironment	(109)
The anti-PD-L1 antibody is conjugated with β-G	Dendritic cells	Promote the interaction between tumor cells and dendritic cells and inhibit the occurrence of CRC	(89)
influenza	Macrophages infiltrate the tumor lesion	Mucosal alveolar macrophages trained for influenza exhibit powerful phagocytic and cytotoxic effects	(75)

### 2.2 TI and macrophages

Macrophages played a central role in TI as their phenotypes and functions dynamically adapted to environmental stimuli. Classically activated M1 macrophages promoted inflammation while alternatively activated M2 macrophages mediated anti-inflammatory responses (32).During TI induction, macrophages underwent significant metabolic shifts from oxidative phosphorylation to aerobic glycolysis (the Warburg effect) when exposed to stimuli like β-G (33). Concurrent epigenetic remodeling occurred not only in tissue macrophages but also in bone marrow myeloid precursors, enabling long-lasting effects (34). Following TI macrophages exhibited enhanced effector functions including improved phagocytosis, increased pathogen clearance, and elevated production of pro-inflammatory cytokines like IL-1β, IL-6 and TNF-α. These cytokines not only directly combated infections but also recruited and activated other immune cells, amplifying the overall immune response. In TI, circulating monocytes serve as the core cells that undergo functional reprogramming. Studies have demonstrated that stimulants such as β-G can induce substantial metabolic and epigenetic changes in human monocytes—for example, an increase in the methylation of the histone marker H3K4me3. Even after these monocytes differentiate into macrophages, this “trained” imprint persists, resulting in a more robust inflammatory cytokine response to secondary stimuli (35). Furthermore, this reprogramming effect extends to monocyte precursors in the bone marrow, thereby exerting a sustained influence on myeloid cells (36).

### 2.3 TI and NK cells

NK cells were identified as another crucial component of the innate immune system capable of developing TI (37). When initially exposed to cytokine stimulation, NK cells rapidly activated intracellular epigenetic modification programs, particularly showing significant histone modifications at the INF-γ gene locus. These epigenetic changes occurred not only in mature NK cells but were also transmitted to daughter cells, establishing a stable “memory imprint.” During TI, NK cells underwent substantial metabolic reprogramming, with glycolytic flux increasing 3-5-fold and lactate dehydrogenase activity enhancing significantly. Mitochondrial alterations included elevated ROS production and improved ATP synthesis efficiency. Functionally, trained NK cells demonstrated approximately threefold increases in perforin and granzyme expression, substantially enhanced cytotoxic capacity, and markedly elevated production of cytokines including INF-γ and TNF-α. This trained resulted in more potent and rapid anti-infection responses (38). Clinical observations revealed that MMR (Measles Mumps Rubella) vaccines could induce long-term immune memory, with transcriptomic analyses showing changes in both CD14^+^ monocytes and NK cells, though the most pronounced effects were observed in γδT cells (39).

### 2.4 TI and ILCs

TI and ILCs are both key components in the field of innate immunity. They engage in cross-functional collaboration and work together to enhance the body’s defensive capacity against threats such as pathogens and tumors. In the human immune system, innate immunity and adaptive immunity work in concert to fend off pathogen invasion. ILCs can be activated without antigen presentation and thus mount rapid responses to signals from pathogens or tumors (40).

Emerging evidence highlights the potential of TI in conferring protection against diverse pathogens. A pivotal study demonstrated that replication-deficient human adenovirus vectors can effectively TI system, resulting in protective immunity in mice against a broad spectrum of influenza virus strains, including H1N1, H3N2, H5N2, H7N9, and H9N2. Notably, bovine and chimpanzee adenoviruses were also capable of activating human ILCs and provided protection against H3N2 influenza challenge in mice. A critical finding was that this protection occurred independently of the conventional influenza-specific adaptive immune response, as evidenced by the absence of hemagglutination-inhibiting antibodies, neutralizing antibodies, and influenza nucleoprotein-specific CD8^+^T cells. The underlying protective mechanisms were identified as the enhanced activation of ILC1, ILC2, and ILC3 populations, increased expression of interferon-stimulated genes (ISGs), upregulation of antiviral signaling pathways, and metabolic reprogramming within ILC subsets. This research establishes a novel strategy for bolstering innate antiviral immunity, offering a promising approach not only for epidemic preparedness against known influenza strains but also as a countermeasure against emerging infectious diseases (41).Clinical investigations into allergen-specific immunotherapy (AIT) have revealed its profound impact on the innate immune compartment. Observational clinical studies indicate that AIT induces significant changes in the composition and heterogeneity of circulating innate immune cells. Remarkably, these alterations shift the innate immune profile toward a state that mirrors that observed in healthy individuals, suggesting a normalization or resetting of innate immune homeostasis as a component of AIT’s therapeutic mechanism (42).The strategic design of vaccine antigens can critically influence the engagement of innate immunity at mucosal sites, a key battleground for many pathogens. Research on SIV and HIV vaccines revealed that envelope V1-deleted (ΔV1) vaccines, when delivered systemically via the DNA/ALVAC/gp120 platform, conferred a superior reduction in the risk of mucosal SIV or SHIV acquisition compared to their V1-replete counterparts. This enhanced protection was linked to the effective activation of specific innate lymphoid cell populations in the mucosal environment, namely IFN-γ ILC1s and IL-13^+^ ILC2s. This finding underscores the importance of antigen design in eliciting protective mucosal innate immune responses against viral challenges (43). The traditional paradigm of immunological memory as an exclusive feature of adaptive immunity is being challenged by findings in innate lymphoid cells. It has been discovered that stimulation with IL-33 or IL-25, or exposure to the allergen papain, induces the expression of the transcription factor c-Maf in murine group ILC2s. This program enables ILC2s to develop memory-like properties, thereby contributing to the pathophysiology of type 2 inflammatory diseases such as allergies. This phenomenon, often termed “trained immunity” or “innate immune memory” in ILC2s, represents a significant expansion of our understanding of how innate mechanisms can perpetuate and exacerbate chronic inflammatory conditions (44).

### 2.5 TI and γδT cells

Vaccines have long been shown to protect beyond their target antigen through induction of TI via regulating epigenetic, transcriptional, and functional reprogramming of innate immune cells (3, 45, 46). γδT cells represent a unique subset of the immune system, exhibiting characteristics of both innate and adaptive immunity. These cells constitute only 1% to 5% of T cells in peripheral blood, however, when pathogens invade the human body, γδT cells respond with heightened sensitivity, serving as the immune system’s first and faster-acting line of defense. Importantly, γδT cells could combat tumors through multiple pathways, including directly eliminating tumor cells via NK cell receptors and inducing tumor cell apoptosis through apoptosis-related factor ligands (47). A clinical trial using single-cell RNA-Seq has verified that MMR vaccination induces TI via functional and metabolic reprogramming of γδT cells. It is manifested as higher production of TNF and IFN-γ, as well as upregulation of cellular metabolic pathways (39). It is also reported that BCG vaccination has impact on the repertoire of human γδT cell receptors (48). BCG-activation of leukocytes is sufficient for the generation of innate anti-tumor NK and γδT cells (49). And intravenously BCG vaccination and aerosol BCG revaccination could induce mycobacteria-responsive γδT cells immune memory and protect against bacterial infection (50). Interestingly, subcutaneous BCG administration in pre-weaned calves can induce TI in γδT cells. This trained memory presents as increased expression of innate immune response-related genes, thereby inducing a functional TI response evidenced by elevated IL-6 and TNF-α cytokine production (51). Suen TK and their teamwork also confirmed this phenomenon in humans that γδ T cells could serve as key target cells for TI through metabolic and epigenetic regulation, TI can enhance the effector functions of γδ T cells and even extend the duration of their “memory-like state” (52). Similarly, another recent study confirmed that respiratory syncytial virus infection confers heterologous protection against SARS-CoV-2 via induction of γδ T cell-mediated TI (53). It means that TI could provide a long-term cross-protection against various infections.

### 2.6 TI and neutrophils

As mentioned earlier, neutrophils are the most abundant leukocytes which have multiple functions during immune response and the first responders when challenged with infection or inflammation diseases (54, 55). In addition, neutrophils have been recently identified as vital effectors of peripheral TI, they may exert beneficial effects in infection and cancer diseases (56). It is reported that the absolute number of neutrophils increases signally following BCG vaccination in healthy humans and these neutrophils remain in a trained state after 3 months. More importantly, the capacity of degranulation and phagocytic as well as IL-8 and ROS production in these trained neutrophils have all elevated when facing with the secondary stimulus (57). Research also indicated that TI participated in the progression of diabetes by regulating the formation of extracellular trap in neutrophils (58). Another study has confirmed that BCG and β-G could reprogram the hematopoietic stem and progenitor cells in innate immune cells, especially neutrophils, these trained neutrophils showed higher ROS production and tumor core infiltration to restrain the vascularization and tumor progression (59, 60). Meanwhile, BCG-trained neutrophils could reshape the tumor microenvironment to drive the adaptive anti-tumor immune responses and augment the effect of immune checkpoint blockade therapy (61). All above foundation suggests that TI maybe beneficial to the solid cancer therapy. For further, BCG vaccination could amplify the granulocytic-macrophage progenitors and enhance their oxidative metabolism as well as the ability which stimulates T cell proliferation to improve neonates’ survival to polymicrobial sepsis (62). In a clinical trial, researchers have indicated that the frequency of eosinophils, neutrophils, NK cells and donor-unrestricted T cells all increased 7 days after aerosol BCG infection (63). It is worth noting that the innate immunity elicited by BCG could be better control the SARS-CoV-2 replication and this inhibition is related to higher frequencies of lung infiltrating neutrophils and higher CD14^+^ cells (64). Likewise, prior low dose of lipopolysaccharide (LPS) exposure-induced TI could upregulate the expression of antibacterial protein, galectin-3 in neutrophils to protects mice from a lethal bacterial infection (65). All above reviewed findings indicated that trained-neutrophils participate in the progression of multiple infection and cancer diseases, manipulating TI in neutrophils may enhance the function of neutrophil and provide a strategy to treat infection and cancers.

## 3 Research on TI in cancer

TI played an indispensable role in many diseases, though not all manifestations of TI were beneficial to health. For example, in atherosclerotic cardiovascular disease (ASCVD), TI was identified as a potential driver of chronic inflammation. Studies suggested that endogenous pro-atherosclerotic factors could induce TI, triggering epigenetic reprogramming in immune cells. This implied that TI might activate cardiovascular disease mechanisms through systemic modulation of bone marrow hematopoietic progenitor cells (66). While TI exhibited detrimental effects in ASCVD, it demonstrated protective roles in oncology. In tumors, TI enhanced immune responses, suppressed metastasis, and prolonged survival. β-G-induced TI elicited stronger responses to LPS and amplified reactivity to tumor-derived factors. TI also showed therapeutic potential in chronic disease recovery, muscle injury repair, and tissue regeneration. For instance, β-G stimulation modulated gut microbiota, improved lipid/glucose metabolism, reduced cholesterol, and conferred protection against microbial infections—effects relevant to metabolic syndrome and gastrointestinal disease management (67). Further evidence revealed that WGP(whole beta-glucan particle) induced TI inhibited lung metastasis and boosted anti-tumor activity. Nano-biologics and β-G-induced TI were reported to exert anti-tumor effects in primary subcutaneous tumors. WGP-trained macrophages exhibited a “trained” response upon exposure to tumor cells or tumor-derived factors, suggesting a role in immune surveillance against tumor progression and metastasis (68) (**Table 1**).

### 3.1 Lung cancer

**Figure 1:**
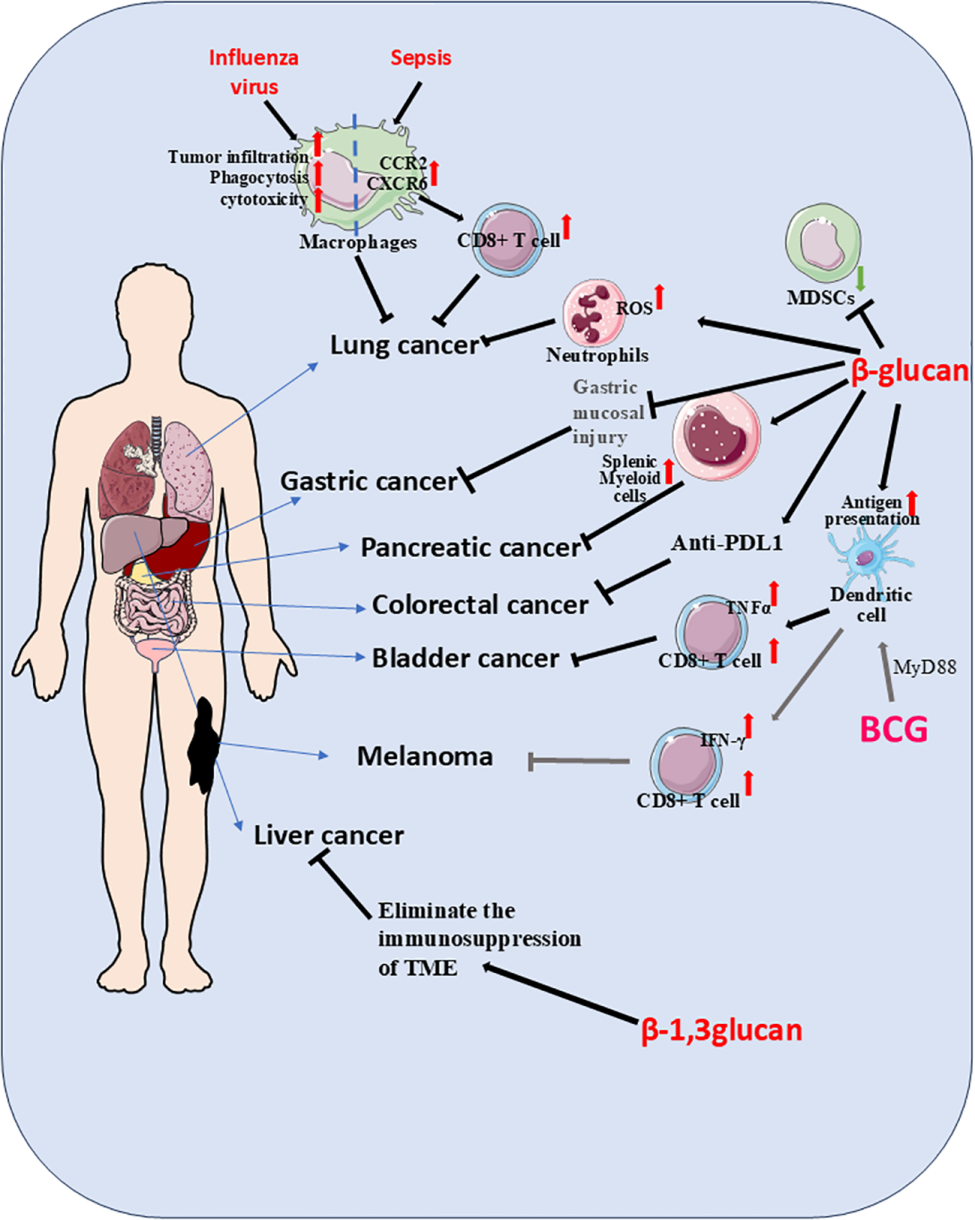
Stimulation with trained immunity (TI) inducers such as β-G or BCG not only activated innate immunity but also enhanced adaptive immune responses. During secondary challenges, TI potentiated the capacity of immune cells to recognize and eliminate malignant cells. In lung cancer models, β-G-stimulated neutrophils suppressed tumor growth through reactive oxygen species (ROS)-dependent mechanisms, while influenza virus-primed TI macrophages infiltrated tumor sites, exhibiting potent phagocytic and cytotoxic activity. Pulmonary macrophages in TI states following sepsis released CCR2 and CXCR6 chemokines, promoting T-cell tissue residency and subsequent tumor inhibition. For pancreatic cancer, nanobiologic targeting of splenic myeloid cells induced peripheral TI, establishing robust anti-tumor immunity. Intravenous BCG administration fostered a bladder tumor-suppressive microenvironment characterized by elevated cytotoxic T lymphocyte (CTL)/TNFα ratios and reduced myeloid-derived suppressor cell (MDSC) populations. TI-conditioned dendritic cells demonstrated enhanced antigen capture and presentation capabilities, significantly amplifying CTL proliferation. BCG-induced TI played a pivotal role in bladder cancer immunotherapy, where MyD88 signaling emerged as critical for mediating anti-melanoma effects. Through the MyD88 pathway, BCG not only generated tumor-specific immunity but also promoted dendritic cell-mediated TI, ultimately enhancing CD8+ T cell anti-tumor responses. The combined treatment of β-G and anti-PD-L1 can significantly inhibit the growth of CRC. β-1, 3-glucan can eliminate the immunosuppression of the tumor microenvironment, thereby slowing down the progression of liver cancer. When β-G is used in combination with drugs as a carrier, it can reduce gastric mucosal damage and more effectively alleviate precancerous lesions.

Lung cancer represented a heterogeneous pulmonary malignancy with diverse clinicopathological characteristics, broadly categorized as small cell lung cancer (SCLC) or non-small cell lung cancer (NSCLC), the latter further divisible into squamous and non-squamous subtypes (69). As the leading cause of cancer mortality in many developed countries, its primary etiology was tobacco smoking - in high-smoking prevalence nations, over 80% of lung cancer cases were smoking-related (70). Statistical data showed lung cancer deaths in Canada exceeded combined mortality from colorectal, pancreatic, and breast cancers (71). Projections indicated the global cancer burden would double by 2050 compared to 2020 levels, with lung cancer remaining predominant. The disease’s high mortality stemmed from frequent late-stage diagnosis and elevated risk among populations exposed to smoke, occupational hazards (e.g., oil fields), and tobacco use. However, advances in screening techniques and novel therapies had begun reducing lung cancer mortality rates. Studies demonstrated that lung-resident alveolar macrophages which underwent TI exhibited enhanced resistance to pathogen-induced cell death. These cells effectively prevented excessive tissue inflammation and rapidly restored homeostasis following repeated pathogen exposure (7). Relevant research confirmed that TI significantly bolstered anti-lung cancer defenses by promoting M1-like macrophage polarization within tumor tissues (72). In murine models, β-G-induced TI enabled neutrophils to suppress lung tumor growth through a ROS-dependent mechanism (73) (**Figure 1**, **Table 1**). The induction of macrophage-mediated TI emerged as a promising strategy for cancer prevention and treatment. For lung cancer specifically, BCG-disguised macrophage membranes demonstrated selective tumor-targeting capabilities (74) (**Table 1**).Intravenous BCG administration synergistically enhanced immune checkpoint-blocked anti-tumor activity without systemic toxicity, highlighting TI’s exceptional anti-tumor potential. Notably, influenza-trained mucosal alveolar macrophages established long-term pulmonary anti-tumor immunity. In murine models of influenza and lung metastasis, these TI-activated macrophages infiltrated tumor lesions, exhibiting potent phagocytic and cytotoxic effects (75) (**Table 1**). Sepsis-trained lung macrophages stimulated CCR2 and CXCR6 chemokine release, triggering T-cell tissue residency - an immune mechanism that reduced post-sepsis tumor recurrence risk. This suggested sepsis-induced TI conferred protective effects against lung cancer development (76). The incorporation of β-G into alginate hydrogel-based nanovaccine formulations facilitates targeted delivery to lymph nodes and dendritic cells (DCs). This specific targeting significantly promotes antigen uptake and maturation of DCs, accompanied by enhanced production of pro-inflammatory cytokines, including IL-1β, IL-6, and TNF-α. In murine models, this nanovaccine strategy resulted in the complete suppression of TC-1 tumor growth and, notably, the eradication of established lung tumors (77) (**Table 1**). A complex clinical interplay exists between lung cancer and active tuberculosis (TB), wherein certain anticancer agents can independently promote TB progression. For instance, the chemotherapeutic drug Gemcitabine (Gem) induces granulocyte-biased hematopoiesis in the bone marrow via G-CSF signaling, leading to neutrophilic pulmonary inflammation. However, pre-existing *Mycobacterium tuberculosis*-specific T cell responses, such as those elicited by BCG vaccination, can normalize granulopoiesis by restricting G-CSF production. This immunomodulatory effect consequently suppresses lung cancer development (78).

### 3.2 Colorectal cancer

Colorectal cancer (CRC) represents a significant global health burden as the fourth leading cause of cancer mortality worldwide, accounting for 9.2% of all cancer-related deaths. This malignancy exhibits notable gender disparities, ranking as the second most common cancer in women and third in men, with male incidence and mortality rates consistently 25% higher than females. The epidemiology of CRC further reveals substantial racial variations, with non-Hispanic Black populations experiencing the highest disease burden while Asian Americans/Pacific Islanders demonstrate the lowest prevalence. Although incidence rates have historically declined in individuals over 50 years old, recent epidemiological data indicates a concerning reversal of this trend in this age group (79). The development of CRC involves a complex interplay between environmental factors and genetic predisposition, with clinical manifestations categorized into hereditary, familial, and sporadic forms (80). Dietary patterns, particularly fiber consumption, have been strongly associated with disease risk. Mounting scientific evidence has elucidated the critical role of intestinal microbiota composition in CRC pathogenesis, establishing specific microbial profiles as potential diagnostic biomarkers (81).The human gastrointestinal tract hosts an extraordinarily diverse microbial ecosystem consisting of over 1000 species encompassing 1014 distinct microbial types (82). These commensal microorganisms perform essential physiological functions including maintaining intestinal homeostasis (83), regulating energy metabolism (84), reinforcing epithelial barrier integrity, and enhancing host defense mechanisms against pathogenic invaders (85). Contemporary CRC treatment strategies include radiotherapy, which has been demonstrated to significantly alter gut microbiota composition, particularly through the depletion of beneficial symbiotic bacteria such as Bifidobacterium and Clostridium species (86). Beyond conventional approaches, emerging therapeutic modalities in regenerative medicine, particularly stem cell therapy, have shown considerable promise in the management of CRC (87).

Of note, Dectin-1 (encoded by the gene *Clec7a*), a known receptor for β-G, plays a pivotal role in host defense against fungi and the maintenance of intestinal immune homeostasis. Intervention with the short-chain β-G laminarin, a Dectin-1 antagonist, has been demonstrated to suppress the development of colorectal tumors in murine models. Mechanistically, Dectin-1 signaling upregulates the expression of prostaglandin E2 (PGE2) synthases, and subsequently, PGE2 inhibits the expression of IL22RA2 in human CRC-infiltrating cells. These observations collectively suggest the existence of a Dectin-1–PGE2–IL-22BP axis that modulates intestinal tumorigenesis, thereby positioning Dectin-1 as a potential therapeutic target for CRC (88). Building upon the immunomodulatory potential of β-G, a novel strategy involving the conjugation of an anti-PD-L1 antibody with β-G has been developed. This construct is designed to promote interactions between tumor cells and DCs, thereby augmenting the benefits of immunotherapy. In the MC38 murine model of colorectal cancer, this conjugated agent exhibited a potent antitumor effect, achieving a remarkable tumor suppression rate of 86.7% (89) (**Table 1**). Oat-derived β-G, a soluble dietary fiber, possesses a broad spectrum of bioactivities, including anti-inflammatory and anti-tumor properties. Research indicates that its suppressive effect on colorectal cancer development in rats is mediated through the stimulation of both autophagy and the extrinsic apoptosis pathway (90). Lentinan (LNT), a β-D-glucan purified from the cultured fruiting body of *Lentinula edodes* mushrooms, also exhibits significant antitumor efficacy. In cervical cancer, LNT exerts its inhibitory effects by activating the tumor suppressor p53, leading to cell proliferation arrest and apoptosis via the p21 and mitochondrial-dependent pathways (91) (**Table 1**). Furthermore, molecular size reduction of LNT through specific processing enhances its bioactivity against colorectal cancer, enabling the suppression of cancer cell proliferation, induction of apoptosis, and reduction of inflammation (92).

### 3.3 Breast cancer

Breast cancer represents a complex and heterogeneous malignancy influenced by both genetic predisposition and environmental factors. As the most prevalent life-threatening cancer among women in Western nations, it ranks as the second leading cause of female cancer mortality, surpassed only by lung cancer (93). The aggressive nature of breast cancer stems largely from cancer stem cells, which drive tumor invasiveness and metastatic potential (94). Epidemiologically, the disease affects approximately 1 in 20 women globally (95), and holds the distinction of being the most frequently diagnosed malignancy during pregnancy (96). While predominantly affecting women, breast cancer incidence in men has shown a steady increase worldwide. Key risk factors for male breast cancer include obesity, testicular disorders, other primary tumors, and particularly BRCA2 gene mutations, with carriers facing an 80-fold greater risk compared to the general population (97). Histologically, invasive lobular carcinoma constitutes the second most common subtype after invasive ductal carcinoma, representing 10-15% of all cases. These tumors typically exhibit luminal molecular features with estrogen and progesterone receptor positivity and HER2 negativity, contributing to their unpredictable response to neoadjuvant treatments (98). Among the most aggressive variants, metaplastic breast cancer accounts for less than 1% of invasive cases. This rare subtype demonstrates distinct pathological characteristics, including adenocarcinoma Tous differentiation with spindle cell, squamous epithelial, and/or mesenchymal components. Clinically, it shows marked chemotherapy resistance and carries a particularly poor prognosis with low survival rates (99). The evolution of breast cancer management has progressed significantly from radical mastectomy to modified radical procedures and currently favors breast-conserving approaches incorporating chemotherapy and radiotherapy (100). However, the emergence of drug resistance in advanced disease poses a major therapeutic challenge, primarily driven by endocrine dysfunction and hormonal imbalances (101). Studies have demonstrated that the number of γδT cells in breast tissue is closely associated with the survival duration of breast cancer patients: the higher the number of these cells, the longer the patients’ survival time. This finding also provides a novel immunotherapeutic strategy for intervening in triple-negative breast cancer. Recent investigations highlight the significant potential of particulate β-1,3-glucans in modulating tumor biology and enhancing chemotherapeutic efficacy, particularly in the context of breast cancer. One such agent, a granular β-1,3-glucan extracted from the spore wall of *Ganoderma lucidum* (GPG), has been identified as a biological response modifier. Notably, research demonstrates that GPG can reverse the immunosuppressive tumor microenvironment (TME) induced by Gem chemotherapy. This reversal is characterized by the mitigation of GEM-induced reductions in anti-tumor T cells and the suppression of increases in both myeloid-derived suppressor cells (MDSCs) and regulatory T cells (Tregs) (102) (**Table 1**). Concurrently, strategies to ameliorate the dose-limiting cytotoxicity of conventional chemotherapeutics, such as doxorubicin (DOX), have leveraged the unique properties of yeast-derived β-G particles (YGPs). YGPs possess a hollow, porous vesicular structure, making them excellent candidates for drug delivery. For instance, DOX encapsulated within YGPs has shown potent cytotoxic effects against various breast cancer cell lines, including MCF-7 and 4T1, while simultaneously reducing the associated physiological toxicity of the free drug (103). Further advancing this concept, β-G-based nanotubes have been engineered as promising nanocarriers for the delivery of hydrophobic DOX. This formulation has demonstrated superior efficacy compared to free DOX, achieving a 74.5% inhibition rate in breast cancer models. It promotes tumor cell apoptosis, blocks proliferation, and results in significantly suppressed tumor growth both *in vitro* and *in vivo*, alongside a marked reduction in adverse effects (104).

The therapeutic application of β-G-based drug delivery systems can be further potentiated by combination therapies. A related study indicates that a similar β-G nanoplatform, when co-administered with photothermal therapy, can effectively block the synthesis of essential nutrients required for tumor DNA replication. This combinatorial approach significantly induces oxidative stress and ultimately exacerbates DNA damage in breast cancer cells, leading to enhanced therapeutic outcomes (105) (**Table 1**).

### 3.4 Bladder cancer

Bladder cancer represents the most prevalent and lethal malignancy of the urinary tract system, with lymphatic spread serving as the predominant metastatic pathway (106). Annually, this disease is diagnosed in over 430000 individuals globally and accounts for approximately 170000 deaths, underscoring its significant public health impact. Epidemiological studies reveal pronounced gender disparities in incidence patterns, with emerging evidence suggesting androgen receptor signaling plays a pivotal role in driving tumor development, disease progression, and clinical recurrence (107). Among established risk factors, tobacco smoking remains the most consistently identified environmental contributor to bladder carcinogenesis (108). The standard immunotherapy for patients with non-muscle-invasive bladder cancer involves intravesical instillations of BCG. However, the precise contribution of TI to BCG’s mechanism of action in suppressing bladder cancer progression remains to be rigorously quantified and warrants further careful investigation (59). BCG, a live attenuated vaccine derived from *Mycobacterium bovis* and primarily used against tuberculosis, also serves as an immunotherapeutic agent in oncology. A significant clinical limitation is its requirement for repeated administrations and its failure to elicit a response in approximately 50% of patients (59). Intriguingly, preclinical studies using an orthotopic bladder cancer model have demonstrated that a single intravesical dose of BCG, when combined with β-G, can lead to the eradication of invasive tumors with a 100% survival rate. In this context, reprogrammed neutrophils exhibited enhanced ROS production and infiltrated the tumor core, resulting in reduced tumor vascularization and growth. The concept of TI appears to play a pivotal role in remodeling the tumor immune microenvironment. For instance, macrophages trained with BCG, upon co-administration with ovalbumin-expressing bladder tumor cells, were shown to increase the proportion of tumor-specific cytotoxic T lymphocytes (CTLs) (109) (**Table 1**). Furthermore, BCG-trained dendritic cells demonstrate enhanced antigen uptake and presentation, subsequently promoting the proliferation of CTLs (109) (**Table 1**). The systemic immunomodulatory effects of BCG are also evident at the level of hematopoiesis. While intravenous administration of BCG is known to reprogram hematopoietic stem and progenitor cells (HSPCs) to confer heterologous protection against infections, intravesical administration can also lead to BCG colonization of the bone marrow. In both murine models and humans, this reprograms HSPCs to alter and expand myelopoiesis. These BCG-reprogrammed HSPCs are sufficient to enhance anti-bladder tumor immunity by generating neutrophils, monocytes, and dendritic cells (61).The combination of BCG therapy with molecular interventions, such as Mcl-1 shRNA, has been shown to shift macrophage polarization towards an anti-tumor M1 phenotype. This is characterized by elevated expression of M1 markers (TNF-α, CD86, iNOS) and reduced expression of M2 markers (IL-10, CD206, Arg-1), ultimately leading to significantly increased apoptosis of bladder cancer cells and diminished proliferative, migratory, and invasive capacities (110) (**Table 1**).Concurrently, numerous studies are exploring the development of novel BCG-based composites to augment its anti-tumor potential. For example, innovative drug delivery strategies have been employed, such as a “biotin-streptavidin strategy” to coat live BCG with nanoparticles loaded with the chemotherapeutic agent DOX. This approach has proven effective in inhibiting cancer progression and prolonging survival in rat/mouse models of orthotopic bladder cancer. Notably, these constructs also demonstrated improved tolerability, biosafety, and the establishment of a potent anti-tumor immune response within the tumor microenvironment (111).The tumor microenvironment in bladder cancer is notably enriched with tumor-associated macrophages (TAMs), which represent one of the most abundant immune cell populations infiltrating these malignancies. While TAM-targeted therapies have shown promising results in other cancer types, this approach remains under investigated in bladder cancer (112).Molecular characterization of bladder cancer has identified several recurrent genetic alterations, including mutations in TERT promoter regions, FGFR3, TP53, PIK3CA, and STAG2 genes, along with various chromatin-modifying genes (113). For patients with high-risk non-metastatic disease, ranging from high-grade non-muscle invasive to muscle-invasive presentations, radical cystectomy with lymph node dissection continues to serve as the gold standard therapeutic approach (114). The BCG vaccine induced stronger TI, which was characterized by an enhanced response to pro-inflammatory factors, greater reprogramming of myeloid cells toward inflammatory and activated states, and more significant epigenetic and metabolomic alterations. In bladder cancer models, the BCG vaccine was found to boost immune activity and exert anti-tumor effects (16). Another study demonstrated that intravenous administration of the BCG vaccine promoted an anti-tumor bladder microenvironment, evidenced by an increased proportion of CTLs and a decreased proportion of MDSCs (**Figure 1**). This TI-mediated remodeling of the tumor immune microenvironment played a crucial role in driving anti-tumor immunity (109). Intravenous injection of the BCG vaccine created an anti-tumor bladder microenvironment, specifically marked by a rise in CTLs and a reduction in MDSCs (115). Following either intravenous or intravesical BCG administration, systemic anti-tumor immunity became polarized, with TI contributing to the reshaping of the tumor immune microenvironment. When TI-primed macrophages were co-cultured with ovalbumin-expressing bladder tumor cells, an increase in tumor-specific CTLs was observed. Furthermore, dendritic cells conditioned by TI displayed stronger antigen uptake and presentation capabilities, enhancing CTL proliferation. These findings collectively indicate that BCG-induced TI plays a significant role in mediating anti-tumor immune responses in bladder cancer (109).

### 3.5 Pancreatic cancer

Pancreatic cancer remains one of the most aggressive malignancies, with a dismal prognosis characterized by a median survival of just 4 months and a 5-year survival rate hovering around 10% (116). The disease demonstrates distinct geographic variation, with the highest incidence rates observed in North America, Europe, and Australia. The rising global incidence is largely attributable to population aging, with additional risk factors including tobacco use, obesity, diabetes mellitus, and excessive alcohol consumption (117).While exceptionally rare in pediatric populations, childhood cancers - particularly renal cell carcinoma - may metastasize to the pancreas, sometimes manifesting more than two decades after initial diagnosis (118). The overwhelming majority of patients present with advanced disease, leaving few viable treatment options and contributing to the characteristically poor outcomes (119). Those with familial pancreatic cancer syndromes or specific genetic mutations face substantially elevated risks of developing the disease. Emerging research has identified long non-coding RNAs (lncRNAs) - including MACC1-AS1, LINC00976, LINC00462, LINC01559, HOXA-AS2, LINC00152, TP73-AS1, XIST, SNHG12, LUCAT1 and UCA1 - as key regulators of pancreatic cancer progression (120). Current projections suggest pancreatic cancer will become the second leading cause of cancer mortality by 2030. Clinical staging categorizes disease into three main groups: resettable, borderline resettable/locally advanced, and metastatic (121). The tumor’s characteristic immunologically “cold” microenvironment renders it particularly refractory to immunotherapy approaches (122), with similarly poor responses observed to targeted therapies, chemotherapy, and radiation (123). Current surgical management may involve neoadjuvant therapy followed by procedures such as pancreaticoduodenectomy, extended pancreatectomy, and lymphadenectomy (124). Emerging evidence highlights the promising role of systemic β-G administration in reprogramming innate and adaptive immunity to combat pancreatic ductal adenocarcinoma (PDAC). In a murine model resistant to checkpoint inhibition, concurrent activation of Dectin-1 via β-G and CD40 using an agonist antibody drives T cell–mediated IFN-γ signaling. This synergistic signaling cascade reprograms distinct macrophage subsets to promote antitumor responses, leading to the eradication of established tumors and the induction of long-term immunological memory (125). Furthermore, β-G has been demonstrated to activate liver-resident macrophages (Kupffer cells) in murine cancer models, promoting their inflammatory polarization. This activation not only suppresses the proliferation of pancreatic cancer cells but also elicits a potent T cell–dependent inhibitory response against hepatic metastases of pancreatic cancer (126). Another innovative approach involves combining β-G–induced TI in peripheral monocytes with non-thermal tumor ablation via IRE. Studies showed that yeast-derived β-G effectively induced TI, leading to a significant reduction in pancreatic tumor burden in murine models. When administered in combination with irreversible electroporation, β-G treatment substantially enhanced TI-mediated anti-tumor responses against pancreatic cancer and prolonged survival in tumor-bearing mice (31). In an orthotopic pancreatic cancer model, this combination therapy significantly reduced both local and distant tumor burden and extended survival, underscoring the potential of integrating innate immune training with physical ablation techniques (31). Beyond irreversible electroporation, pancreatic cancer recurrence was also prevented through nano-biologic targeting of splenic myeloid cells, which induced peripheral TI to mount an effective anti-tumor defense (127) (**Figure 1**).

### 3.6 Melanoma

Melanoma, a malignant tumor arising from melanocytes, developed either *de novo* or within pre-existing moles, frequently demonstrating cutaneous and subcutaneous metastatic potential during disease progression (128). In subcutaneous melanoma models, BCG vaccine stimulation of murine melanoma cells demonstrated the critical role of MyD88 signaling in mediating BCG-induced immunotherapy. The absence of MyD88 in knockout models prevented TI-mediated inflammatory cell recruitment within the tumor microenvironment, consequently impairing tumor suppression and T cell-derived INF-γ production, collectively establishing the essential role of the MyD88 pathway in BCG’s anti-tumor immunity (129) (**Figure 1**). Investigations into squalene cyclooxygenase, a pivotal TI-regulating enzyme, revealed its significant contribution to TI-mediated anti-tumor responses (130). Complementary studies demonstrated that cholera toxin B subunit-induced TI in dendritic cells effectively potentiated CD8^+^ T cell anti-melanoma immunity in murine models (131). Cancer vaccines hold significant potential in clinical oncology by eliciting T cell-mediated immunity. However, their efficacy is often severely compromised by several key limitations: a restricted number of antigen-presenting cells (APCs) at the injection site, insufficient phagocytosis of tumor antigens by APCs, and the potent immunosuppressive tumor microenvironment (72). Emerging strategies to overcome these barriers involve the use of β-G to induce TI. For instance, engineered β-G formulations are highly accumulated and phagocytosed by macrophages at the injection site. This uptake initiates a state of TI, leading to a robust adaptive anti-tumor immune response and the establishment of long-term immunological memory. This approach has demonstrated potent prophylactic and therapeutic efficacy, effectively inhibiting melanoma growth *in vivo (*72). The promise of β-G-induced TI extends to the control of metastasis, a leading cause of cancer-related mortality where myeloid cells play a critical role. Studies show that WGP can reprogram macrophages, enhancing their reactivity not only to lipopolysaccharide but also to tumor-derived factors. This trained phenotype resulted in reduced lung metastasis of melanoma and prolonged survival in multiple murine metastatic models (68). Similarly, intraperitoneal administration of β-G in combination with IFN-γ was found to expand a novel subset of immunostimulatory IL27^+^ macrophages, inducing potent tumor regression in a clinically relevant model of metastatic ovarian cancer (132). It is important to note that the efficacy of TI may be modulated by systemic metabolites. Recent evidence indicates that trimethylamine N-oxide (TMAO), a gut microbiota-derived metabolite, can augment β-G-induced TI. This interaction suggests new therapeutic targets not only for cancer but also for a range of conditions including cardiovascular diseases (CVD), CKD-promoted CVD, inflammation, transplantation, and aging (133). Further supporting the therapeutic application of these molecules, synthetic, water-soluble β-G (molecular weight~60 kDa) have been developed. These compounds significantly inhibit the migration of highly metastatic melanoma B16F10 cells *in vitro*. Notably, *in vivo* administration substantially reduced and eliminated tumor nodules, achieving a remarkable inhibition rate of 86.7% (134). Moreover, with the deepening of melanoma research, TI holds promising potential for more effective combination with other treatment modalities, which will in turn enhance therapeutic outcomes to a greater extent.

### 3.7 Liver cancer, gastric cancer and other cancers

Primary liver cancer represents the third most prevalent cause of cancer-related mortality globally, characterized by aggressive tumor progression and alarmingly high fatality rates (135). The dismal prognosis primarily stems from late-stage diagnosis, with the majority of cases identified at advanced phases when therapeutic options become severely limited (136). Ranking as the sixth most common primary cancer site in humans, the liver’s distinctive immunosuppressive microenvironment coupled with its unique tissue architecture makes it particularly susceptible to metastatic colonization from other malignancies, especially colorectal cancers (137). β-G, particularly those with specific structural modifications, have emerged as promising immunomodulatory agents in cancer therapy. For instance, surface-modified β-1,3-glucans demonstrate potent immunostimulatory properties and the ability to repolarize macrophages, thereby counteracting immunosuppressive conditions within the tumor microenvironment and suppressing the progression of advanced hepatocellular carcinoma (HCC) (138).

In addition, Photothermal therapy (PTT) and photodynamic therapy (PDT) represent promising modalities; however, their efficacy is often suboptimal when applied individually. Innovative approaches are exploring combinatorial strategies, such as utilizing the photothermal and photosensiting properties of iron diselenide (FeSe_2_). Recent work has evaluated β-G nanotubes loaded with BFP-FeSe_2_ nanocomposites, demonstrating that the combination of PTT and PDT can significantly enhance therapeutic outcomes in HCC (139).

Gastric cancer, a primary epithelial malignancy of stomach origin, presented as a complex heterogeneous disease (140), ranking among the top five most prevalent cancers globally and top three cancer-related deaths. Major risk factors included Helicobacter pylori infection, high salt intake, aging, and low fruit/vegetable consumption (141). In gastric cancer, encapsulating DOX within a mushroom-derived β-G-based carrier mitigates gastric mucosal injury and more effectively alleviates precancerous lesions. This protective and therapeutic effect is achieved through modulation of the p53 and PI3K pathways, leading to reduced oxidative stress and inflammation (142). The translational potential of β-G is further supported by clinical investigations. A preliminary clinical study in patients with advanced gastric adenocarcinoma evaluated a regimen of β-G combined with camrelizumab (an immune checkpoint inhibitor) and SOX chemotherapy (oxaliplatin and oral S-1), administered every three weeks. This combination therapy showed promising efficacy and a manageable safety profile, warranting further validation in larger studies (143).

Beyond gastric cancer, β-G exhibit broad anticancer activity. LNT, a β-D-glucan derived from cultured *Lentinula edoda* mushrooms, disrupts cellular homeostasis in HeLa cervical cancer cells by significantly increasing intracellular Ca^2+^ levels and promoting autophagic flux. This effect is mediated, at least partially, via the PI3K/Akt/mTOR pathway, characterized by upregulation of LC3-II and downregulation of p62, PI3K, phospho-Akt, and mTOR. These molecular changes correlate with inhibited proliferation of HeLa cell xenografts in BALB/c nude mice (144). Interestingly, early-life immune modulation may also influence cancer risk. Epidemiological evidence suggests that childhood BCG vaccination is associated with a significantly reduced incidence of childhood leukaemia (145), hinting at the long-term, systemic immunoprotective effects that certain biological response modifiers can confer.

In summary, β-G demonstrate multifaceted antitumor activities, ranging from direct immunomodulation and sensitization of cancer cells to death pathways, to serving as versatile drug delivery platforms and enhancing the efficacy of combination therapies in clinical settings.

## 4 Future perspectives

The emerging field of TI represents a transformative approach in cancer immunotherapy, offering novel therapeutic strategies through the epigenetic and metabolic reprogramming of innate immune cells. Future investigations should focus on elucidating the precise molecular mechanisms underlying TI’s antitumor effects, particularly the epigenetic modifications (including histone acetylation, methylation patterns, and DNA methylation) and metabolic regulation (especially through mTOR, HIF-1α, and AMPK pathways) that mediate long-term immune memory in various tumor microenvironments. Current advancements in immunomodulatory nanomaterials, particularly β-G-based nanoparticles and innovative nano biotic formulations, demonstrate significant potential to enhance tumor-specific immune responses by improving antigen presentation and T cell activation (146). In the future, it is expected that the existing research bottlenecks will be broken through by optimizing drug delivery strategies (such as using nanoparticle systems to enhance the targeting of inducers), developing new TI agonists, and integrating artificial intelligence technology to analyze the laws of immune regulation. Meanwhile, the single-cell multi-omics approach is expected to provide deeper insights into TI’s cross-organ immune regulatory network, helping to reveal the collaborative regulatory mechanisms among different immune cells. These developments position TI as a highly promising strategy that can not only enhance the efficacy of existing immunotherapies but also overcome tumor drug resistance and prevent recurrence. The research breakthroughs are expected to drive the development of a stronger and more durable framework for tumor immunotherapy, providing new treatment hope for cancer patients. In addition, the development of novel agonists capable of inducing TI represents a major research direction in this field. Current trends indicate a growing focus on combinatorial strategies, such as conjugating glucan with novel biomaterials or targeting antibodies, as well as encapsulating anticancer agents within glucan-based vacuolar structures to achieve sustained release and enhanced targeting. These approaches constitute a promising frontier for therapeutic applications.

It is also important to note that BCG, a well‐established inducer of TI, has long been used as a standard treatment for certain cancers, such as bladder cancer. However, whether the antitumor effects of BCG can be attributed specifically to TI requires careful discrimination and further mechanistic investigation.

Another critical avenue for future research involves the application of high‐throughput sequencing technologies to dissect the impact of TI at the level of specific cell populations or even single cells. Such approaches will help elucidate the fundamental signaling pathways underlying TI and identify more robust biomarkers for its induction and maintenance.

At the same time, the potential of TI to exacerbate or precipitate autoimmune pathologies, as a consequence of its broad‐spectrum immunostimulatory effects, remains a significant concern in the context of cancer therapy. Understanding how to harness its benefits while minimizing such risks represents a key translational challenge.

Nonetheless, as an important functional extension of innate immunity, TI has demonstrated considerable clinical potential and offers a promising outlook for future therapeutic development.

## 5 Conclusion

TI, as an immune memory model formed based on the epigenetic and metabolic reprogramming of innate immune cells, has opened up a brand-new direction for tumor immunotherapy. Its core value lies in establishing long-term and systematic anti-tumor immune memory by inducing epigenetic and metabolic reprogramming of innate immune cells, effectively addressing the main limitations of traditional immunotherapy, such as insufficient improvement of the immunosuppressive state of the tumor microenvironment and short duration of immune memory.

At the cellular level, innate immune cells such as macrophages, NK cells, ILCs and γδT cells undergo metabolic conversion from oxidative phosphorylation to aerobic glycolysis after being stimulated by inducers like β-G and BCG, accompanied by epigenetic remodeling such as histone modifications (such as H3K4me3) and changes in chromatin accessibility. Form a long-term “training imprint”, significantly enhancing phagocytic ability, cytotoxicity and the secretion capacity of pro-inflammatory cytokines such as IL-1β and TNF-α; In cancer applications, TI inducers represented by BCG vaccines have performed outstandingly. They not only enhance the antigen-presenting ability of macrophages and dendritic cells but also reshape the tumor microenvironment. For instance, in bladder cancer models, BCG can promote the infiltration of CTLS and reduce MDSCs. In lung cancer models, β-G can induce pulmonary interstitial macrophages to produce reactive oxygen species to inhibit tumor metastasis. In melanoma studies, the central TI induced by nanobiators can enhance the efficacy of PD-1/CTLA-4 inhibitors - ultimately building a bridge between innate immunity and adaptive immune responses. It exerts anti-tumor effects on various cancers such as lung cancer, gastric cancer, colorectal cancer, liver cancer, breast cancer, bladder cancer, pancreatic cancer and melanoma. Moreover, some induction strategies (such as β-G nanocaterals and recombinant BCG) can synergize with immune checkpoint inhibitors, irreversible electroporation and other therapies.

Most studies are based on animal models, and their clinical transformation effects in different subtypes of human cancers and the screening criteria for applicable populations still need to be verified by more clinical trials.
